# Entomological Investigations on Malaria Vectors in Some War-Torn Areas in the Trincomalee District of Sri Lanka after Settlement of 30-Year Civil Disturbance

**DOI:** 10.1155/2015/367635

**Published:** 2015-02-19

**Authors:** Nayana Gunathilaka, Menaka Hapugoda, Wimaladharma Abeyewickreme, Rajitha Wickremasinghe

**Affiliations:** ^1^Molecular Medicine Unit, Faculty of Medicine, University of Kelaniya, Ragama, Sri Lanka; ^2^Tropical Environmental Diseases & Health Associates, No. 3 Elibank Road, Colombo 5, Sri Lanka; ^3^Department of Parasitology, Faculty of Medicine, University of Kelaniya, Ragama, Sri Lanka; ^4^Department of Public Health, Faculty of Medicine, University of Kelaniya, Ragama, Sri Lanka

## Abstract

*Background.* Malaria was an endemic problem in Trincomalee District, Eastern Province of Sri Lanka. Very few recent data concerning *Anopheles* are available which transmit malaria. Therefore, the aim of this study is to identify various *Anopheles* species and the dynamics of anophelines including malaria vectors in Trincomalee District for effective vector control under the current malaria elimination program embarked in the country. *Method.* Entomological surveys were conducted on a monthly basis, using five entomological techniques, namely, indoor hand collection (HC), window trap collection (WTC), cattle-baited net collection (CBNC), and cattle-baited hut collection (CBHC) from June 2010 to June 2012 in 32 study areas under five entomological sentinel sites. *Results.* Seventeen anopheline species were encountered, of which *Anopheles subpictus* was the predominant species in all sampling methods. It is noted that* A. culicifacies* and *A. subpictus* have adapted to breed in polluted water in urban settings which may cause serious implications on the epidemiology of malaria in the country. *Conclusions.* It is important to determine the abundance, biology, distribution, and relationship with climatic factors of main and secondary malaria vectors in Sri Lanka in order to initiate evidence based controlling programs under the current malaria elimination program in Sri Lanka.

## 1. Background

Malaria was endemic in the Dry Zone of Sri Lanka and was a major public health problem in the country in the past [1]. Chalmers reported the presence of 10 anopheline species in the country by Chalmers [2]. To date, 22 anopheline species have been reported in Sri Lanka [3].* Anopheles culicifacies* was regarded as the only malaria vector in the country till about the early 1980s. Enzyme-linked immunosorbent assay (ELISA) based evidence has shown a large number of anopheline species to be infected with malaria parasites in addition to* A. culicifacies*. These include* Anopheles aconitus*,* Anopheles annularis*,* Anopheles barbirostris*,* Anopheles nigerrimus*,* Anopheles pallidus*,* Anopheles subpictus*,* Anopheles tessellatus*,* Anopheles vagus*, and* Anopheles varuna*. Among these species that have consistently been incriminated as malarial vectors are* A. annularis*,* A. subpictus*,* A. varuna*, and* A. tessellatus* [4]. Determination of risk of malaria transmission requires quick and accurate methods of identification of* Anopheles* mosquitoes, especially when targeting vector control [5].

*Anopheles* mosquitoes breed in areas with water bodies such as ponds, rivers, surface water, wells, and wastewaters [6]. Moreover, these areas are suitable for growth and development of various strains of mosquitoes as ponds, wells, and water bodies of different sizes that are available during rainy seasons [7].

*Anopheles culicifacies*, the primary vector of malaria in Sri Lanka, is known to breed primarily in stream and river systems. However, this species also breeds in other surface water collections and habitats in Sri Lanka [8]. Recent studies have reported that* A. culicifacies* breeds in brackish water bodies [9] and wastewater drains in the Trincomalee District of Sri Lanka [10, 11]. Mathematical models based on the correlation between the abundance of anopheline species and environmental factors such as rainfall, temperature, and humidity may be used to predict vector abundance and thereby malaria epidemics [12].

Over three decades of civil unrest, the conflict situation has had detrimental effects on vector control activities and management of malaria in Trincomalee District, which is an endemic region for malaria in Sri Lanka. With the background that only a few small-scale studies on malaria and its vectors have been reported from this district, a study was designed to explore the current abundance and distribution of malaria vectors in these areas.

Therefore, the aim of this study is to identify various* Anopheles* species and the dynamics of anophelines including malaria vectors in Trincomalee District for effective vector control under the current malaria elimination program embarked in the country.

## 2. Methods

### 2.1. Study Area

Trincomalee District is situated in the Dry Zone of the country within the Eastern Province of Sri Lanka. The district has a land area of 2,727 km^2^ and a population density of 135/km^2^. The average temperature varies from 24.8°C to 30.7°C and the district receives a mean annual rainfall of 1, 649 mm. The district has been traditionally endemic for malaria. However, very few entomological investigations have been carried out for about three decades in the Northern and Eastern Provinces until 2009, due to the terrorist war that took place in the country including this district.

Five sentinel sites, namely, Gomarankadawala, Ichchallampaththu, Mollipothana, Thoppur, and Padavisiripura, were selected for surveillance in consultation with the National Malaria Control Programme. The factors such as past malaria history, availability of breeding sites, an established agricultural community, and feasibility of field operations to collect relevant data were also considered in selecting the study areas.

**Figure 1:**
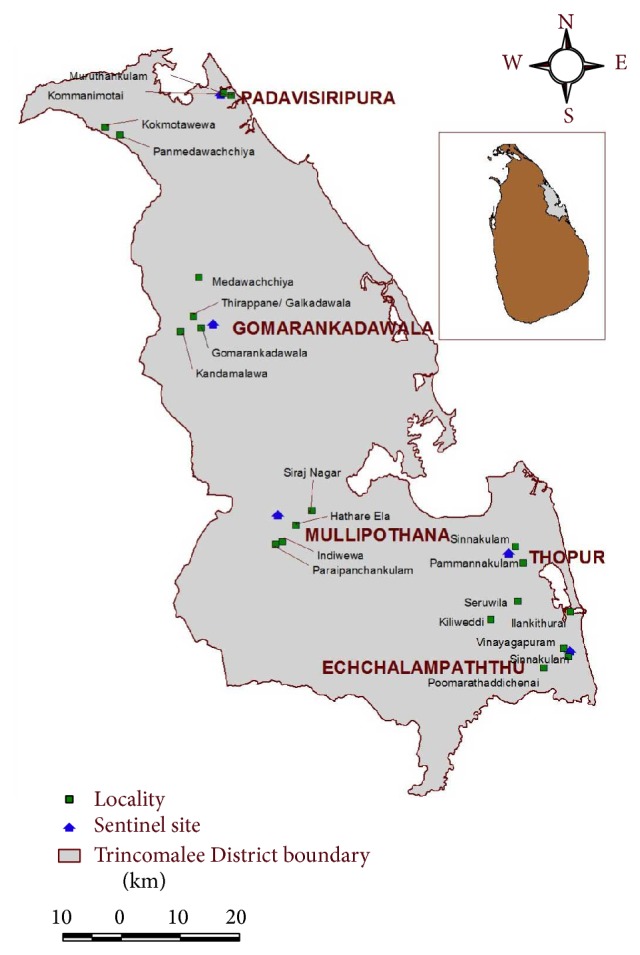
Map showing sentinel sites and localities in Trincomalee District.

The sentinel sites were over 20 Km apart. In each sentinel site, 4 localities were selected within a 20 km radius of the sentinel site. Entomological surveillance was conducted in these 20 localities which lasted 1 week every month (Figure 1).

### 2.2. Mosquito Collection

Mosquitoes were collected at monthly intervals using five standard sampling methods from June 2010 to June 2012 according to WHO standard techniques for anopheline mosquitoes [13].

### 2.3. Adult Anopheline Surveillance

#### 2.3.1. Indoor Hand Collection (HC)

Hand collections were performed in randomly selected houses in each locality. Mosquitoes were collected a minimum of 180 houses per month in a sentinel site. Collections were made during the morning (06.00–08.30 hrs) by two vector collectors spending a maximum of ten minutes per house. Bedrooms, preferably with complete walls and the highest number of persons slept last night were given priority.

#### 2.3.2. Window Trap Collection (WTC)

Two mosquito window (exit) traps were fixed in a sentinel site for 16 nights per month. On the following day mosquitoes were collected by two trained persons.

#### 2.3.3. Cattle-Baited Net Trap Collection (CBNT)

The trap was made out of white cotton drill (3 m × 3 m × 1.5 m) with net windows (2 m × 1 m) on sides and erected using a strong centre pole of two-meter height and four side sticks of the same height. The trap was set about 50 m away from the houses and away from the place, where cattle are usually tethered or herded during the night. A distance of 15–25 cm gap was allowed between the lower edge of the net and the ground, enabling mosquitoes to enter. At sunset a cattle introduced into the trap in the evening and tethered to the pole fixed to the mid of the hut. The cattle removed at dawn for collecting the mosquitoes. All anophelines resting inside the trap were collected.

#### 2.3.4. Cattle-Baited Hut Collection (CBHC)

A standard hut was constructed in each locality. The size of the hut suited the size of the cattle bait and was approximately 2 m × 1.25 m × 1.25 m. It was made of sticks, poles, and thatched with woven cadjan. At sunset, a calf was tethered to a strong pole inside the hut with no windows. A removable door made out of sticks and cadjan was fitted to the hut to facilitate the movement of the calf and the collector in and out of the hut. A space of about 10–15 centimeters (cm) between the ground and the cadjan thatched wall and about a five cm space between the roof and the wall were left for the movement of mosquitoes. All anophelines resting inside the hut were collected on the following day.

#### 2.3.5. Anopheline Larval Surveillance (LS)

All potential breeding habitats were identified in all 20 localities through a preliminary survey conducted for a period of one month prior to the research study and certain fixed and temporary breeding places were identified for the larval survey. A minimum of 50 dips were taken from each breeding habitat depending on the size of the breeding place using standard dippers (250 mL capacity). Large plastic pipettes and small white enamel pans were used for small and shallow water bodies. The* Anopheles* larvae were separated from the Culicine larvae. The* Anopheles* mosquito larvae were classified as early instar stage (I and II) or late instar stage (III and IV) according to Gillies and Coetzee [14].

### 2.4. Sample Identification

All mosquitoes collected by HC, WT, CBNT, and CBHT and adults emerging from larvae were identified using an achromatic magnifying lens (×10) and the taxonomic keys [3].

### 2.5. Calculation of Mosquito Densities

The density of each mosquito species collected by CBHT, CBNT, and WTC was calculated as per trap densities (number of mosquitoes from each species/total number of traps) and HC as density per house (number of mosquito from each species/total number of houses surveyed), and larval densities were calculated as density per 100 dips {(number of mosquitoes from each species/total number of dips) × 100}.

### 2.6. Collection of Climatic Data

Monthly climatic data including rainfall (RF), temperature (MT), and relative humidity (RH) of the Trincomalee District monitored at various locations were obtained from the Department of Meteorology, Colombo, Sri Lanka.

### 2.7. Data Analysis

The data obtained in the study were collected and analyzed with respect to* Anopheles* species abundance in the study area. These were interpreted in percentage and presented in tables. Pearson's correlation coefficients were used to determine the associations between climatic variables and anopheline densities.

### 2.8. Ethical Consideration

Ethical clearance to conduct the study was obtained from the Ethics Review Committee of the Faculty of Medicine, University of Kelaniya, Sri Lanka.

## 3. Results

### 3.1. Mosquito Collections

The overall results of the mosquito collection made by five mosquito sampling techniques named CBHC, CBNC, WTC, HC, and LS during the study period are given in Table 1. A total of 87,710 female mosquitoes representing 17 species were recorded throughout the study. The majority (62%) of adults was collected by cattle-baited collections (CBHC and CBNC) in the district. Larval surveys recorded 24.3% (21,347/87,710) of* Anopheles* from Trincomalee Districts. The density of each mosquito species collected by CBHT, CBNT, and WTC was calculated as per trap densities (number of mosquitoes from each species/total number of traps) and HC as density per house (number of mosquito from each species/total number of houses surveyed) and larval densities were calculated as density per 100 dips {(number of mosquitoes from each species/total number of dips) × 100}.

The most abundant species among the immature and adult collections was* Anopheles subpictus* (26%).* Anopheles culicifacies* accounted for 1.3% of both adult and larval collections. The distribution of mosquitoes collected from different techniques is given in Table 2. The density of* A. culicifacies*,* A. subpictus*, and all anophelines by different surveillance technique is illustrated in Table 3.

### 3.2. Entomological Monitoring

#### 3.2.1. Indoor Hand Collection (HC)

The density of indoor resting anophelines was monitored in selected houses in each locality. A total of 10,626 adults belonging to 13 anopheline species were collected.* A. subpictus* was the most predominant species, while* A. culicifacies* comprised only 0.01% (2/10,626) of all indoor resting mosquitoes. All other anopheline species were present, but in much less densities (<14%).

#### 3.2.2. Window Trap Collection (WTC)

A total of 1,790* Anopheles* representing 12 species were recorded from 3,560 traps performed during the study period.* A. subpictus* (63.85%) was the most abundant species (1,143/1,790), followed by 12.12% (217/1,790) of* Anopheles nigerrimus*, 10.95% (196/1,790) of* Anopheles vagus*, 5.86% (105/1,790) of* Anopheles peditaeniatus*, 3.52% (63/1,790) of* Anopheles barbirostris*, 1.45% (26/1,790) of* Anopheles pallidus*, 0.84% (15/1,790) of* Anopheles varuna*, 0.78% (14/1,790) of* Anopheles annularis*, 0.28% (5/1,790) of* Anopheles barbumbrosus*, 0.22% (4/1,790)* A. culicifacies*, 0.05% (1/1,790) of* Anopheles jamesii*, and 0.05% (1/1,790) of* Anopheles tessellatus.*

#### 3.2.3. Cattle-Baited Net Collection (CBNC)

A total of 42,325 anophelines belonging to 15 species were recorded from 1,621 hut days. The average number of mosquitoes collected per day is 26.11.* A. peditaeniatus*, 42.97% (18,185/42,325), was the most dominant species followed by* A. nigerrimus*, 30.57% (12,938/42,325).

#### 3.2.4. Cattle-Baited Hut Collection (CBHC)

A total of 11,622 anophelines representing 16 species were collected from 1,620 trap-days of collection. The average number of anopheline collected per trap day was 7.2. Among the 16 species collected, the most predominant species was* A. subpictus*, 46.62% (5,419/11,622), followed by 15.26% (1,774/11,622) of* A. nigerrimus*, 10.4% (1,209/11,622) of* A. peditaeniatus*, 9.35% (1,087/11,622) of* A. vagus*, 5.16% (600/11,622) of* A. annularis*, 4.85% (564/11,622) of* A. barbirostris*, 4.48% (521/11,622) of* A. pallidus*, and 1.91% (138/11,622) of* A. jamesii*.

#### 3.2.5. Larval Surveillance (LS)

Immature stages were collected from all types of breeding sites recorded in each locality of the sentinel sites on a monthly basis (Table 3). A total of 21,347 anophelines were recorded representing 15 species from 598,046 dips.* A. subpictus* 24.72% (5,278/21,347) was predominant followed by 24.67% (5,267/21,347) of* A. nigerrimus* and 14.56% (3,109/21,347) of* A. peditaeniatus.* Some species were limited only to selective breeding habitats (Table 4).

### 3.3. Climate Data

**Figure 2:**
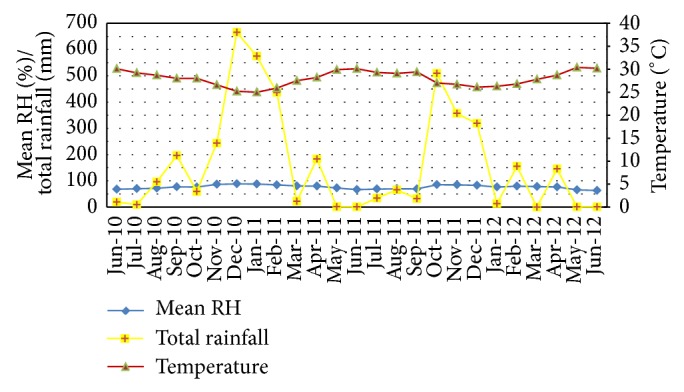
Climatic details of the Trincomalee District during the study period.

The total annual rainfall in 2010 and 2011 was 1376.22 mm and 2532.44 mm, respectively. The highest rainfall was recorded during the months of October to December (Figure 2). The mean relative humidity was 60% during the study period while the mean monthly temperature ranged from 25.0°C to 30.4°C (Figure 2).

### 3.4. Correlations between Anopheline Densities and Climatic Data

The association between both adult and larval anopheline densities and climatic variables, namely, RF, TM, and RH, were investigated by correlation analysis.

#### 3.4.1. Indoor Hand Collection (HC)

*A. culicifacies* density in HC was positively, though not significantly, correlated with RF having a one-month lag period (*r* = 0.25; *P* = 0.23) and a two-month lag period (*r* = 0.39; *P* = 0.063).* A. subpictus* and all anopheline densities in HC were positively, though not significantly, correlated with RF having one-month lag (*r* = 0.05; *P* = 0.81, *r* = 0.087; *P* = 0.69, resp.).

*A. culicifacies*,* A. subpictus*, and all* Anopheles* densities recorded from HC were positively, though not significantly, correlated with RH having a one-month lag period (*r* = 0.28; *P* = 0.15, *r* = 0.21; *P* = 0.35, *r* = 0.24; *P* = 0.27, resp.) and a two-month lag period (*r* = 0.35; *P* = 0.11, *r* = 0.30; *P* = 0.17, *r* = 0.32; *P* = 0.14, resp.). Mean temperature of the current month was positively correlated with* A. subpictus* density in HC (*r* = 0.28; *P* = 0.177) (Table 5).

#### 3.4.2. Window Trap Collection (WTC)

*A. culicifacies* and* A. subpictus* densities were positively, but not significantly, correlated with RF of the current month (*r* = 0.36, 0.29; *P* = 0.07, 0.15, resp.). There was a positive but not significant correlation between RH of the current month,* A. culicifacies* (*r* = 0.242; *P* = 0.245).* A. subpictus* density was positively correlated with RH having a one-month lag period (*r* = 0.399; *P* = 0.054) and a two-month lag period (*r* = 0.389; *P* = 0.067).* A. culicifacies* density was positively correlated (not significant though) with TM having a one-month (*r* = 0.196; *P* = 0.357) and a two-month lag periods (*r* = 0.18; *P* = 0.408) (Table 5).

#### 3.4.3. Cattle-Baited Net Collection (CBNC)

*A. culicifacies* density was positively correlated with RF of the current month (*r* = 0.187; *P* = 0.37), having one-month lag (*r* = 0.25; *P* = 0.25) and two-month lag periods (*r* = 0.33; *P* = 0.125). The RH of the current month (*r* = 0.21; *P* = 0.3), having one-month lag period (*r* = 0.27; *P* = 0.20) and having two-month lag period (*r* = 0.2; *P* = 0.35), was correlated with* A. culicifacies*. Mean temperature of the current month, having one- and two-month lag periods, was negatively correlated with* A. culicifacies* and* A. subpictus* densities (Table 5). None of these correlations were statistically significant.

#### 3.4.4. Cattle-Baited Hut Collection (CBHC)

*A. culicifacies* density was positively correlated, though not significant, with RF of the current month (*r* = 0.066; *P* = 0.75), having one-month lag (*r* = 0.23; *P* = 0.28) and two-month lag periods (*r* = 0.36; *P* = 0.09).* A. subpictus* was positively correlated with the RF of the current month (*r* = 0.31; *P* = 0.31) and RF having two-month lag period (*r* = 0.31; *P* = 0.13). Relative humidity of the current month, having one- and two-month lag periods, was positively correlated with* A. culicifacies* and* A. subpictus* densities (Table 5). None of these correlations were statistically significant. All anophelines densities including* A. culicifacies* and* A. subpictus* densities were negatively correlated, though not significant, with TM of the current month, having one- and two-month lag periods.

#### 3.4.5. Larval Surveillance (LS)

In larval collections,* A. culicifacies*,* A. subpictus*, and all* Anopheles* were positively correlated with the TM of the current month (*r* = 0.18; *P* = 0.39, *r* = 0.53; *P* = 0.007, *r* = 0.30; *P* = 0.14, resp.), having a one-month lag period with* A. culicifacies* (*r* = 0.004; *P* = 0.97) and* A. subpictus* (*r* = 0.30; *P* = 0.15). Larval density of* A. subpictus* was also positively correlated with RH having a two-month lag period (*r* = 0.14; *P* = 0.95). There was a significant negative significant correlation between TM of the current month, having two-month lag periods, with* A. supictus* and all anophelines (Table 5). None of these correlations were statistically significant.

### 3.5. Seasonal Variation of Anophelines

#### 3.5.1. Indoor Hand Collection (HC)

**Figure 3:**
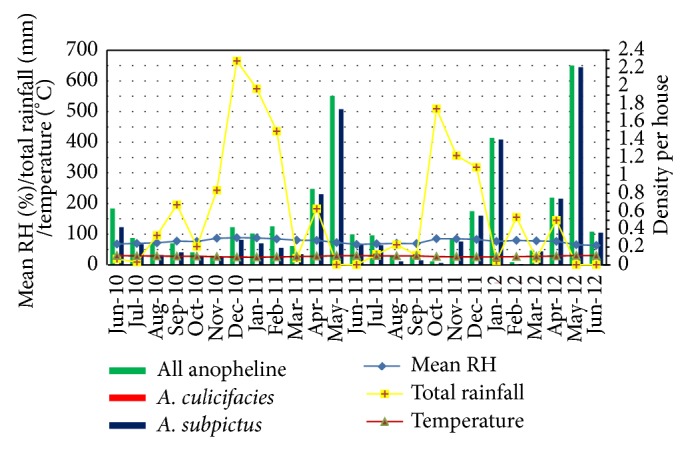
Density of* A. culicifacies*,* A. subpictus*, and all anophelines collected by HC with climatic variables.

The density of all anopheline was observed to be high during the monsoonal rains (May to July and November to January) (Figure 3).* A. subpictus* was the most abundant and only indoor resting species collected throughout the study period.* A. culicifacies* was also collected from Trincomalee District only in December 2011 and January 2012, but very little in numbers when compared to* A. subpictus*.

#### 3.5.2. Window Trap Collection (WTC)

**Figure 4:**
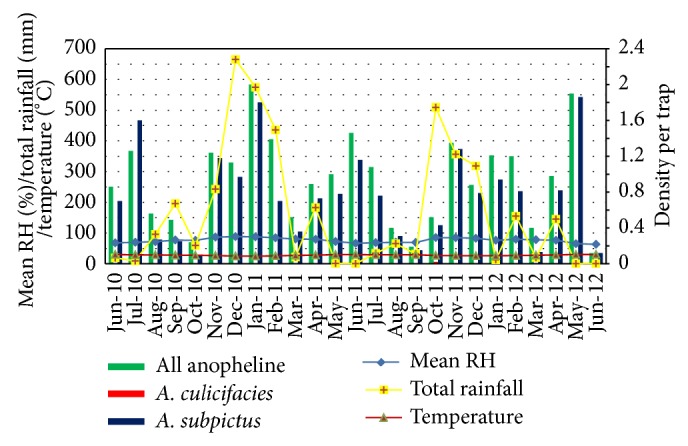
Density of* A. culicifacies*,* A. subpictus*, and all anophelines collected by WTC with climatic variables.

All anopheline density by WTC was high during November to January periods (Figure 4). This is due to the occurrence of monsoonal rains in these seasons. The highest indoor resting densities by WTC peaked approximately one to two months after a high rainfall received for a particular period. The similar pattern was observed for* A. subpictus* also* A. culicifacies* was detected only in October 2011.

#### 3.5.3. Cattle-Baited Net Collection (CBNC)

**Figure 5:**
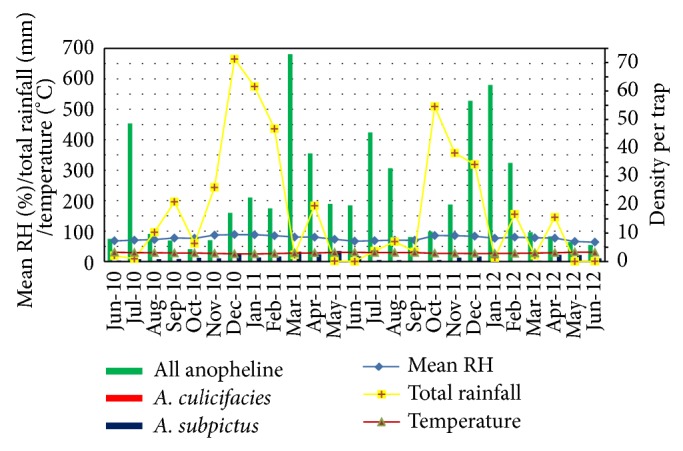
Density of* A. culicifacies*,* A. subpictus*, and all anophelines collected by CBNC with climatic variables.

The highest densities were observed during May to June and November to February (Figure 5). All anopheline density was high in July 2010, March 2011, and January 2012.* A. subpictus* density was observed to be high during December 2010 to May 2011 with increasing rainfall.* A. culicifacies* was found only during November and December 2011. The highest density was observed in December 2011.

#### 3.5.4. Cattle-Baited Hut Collection (CBHC)

**Figure 6:**
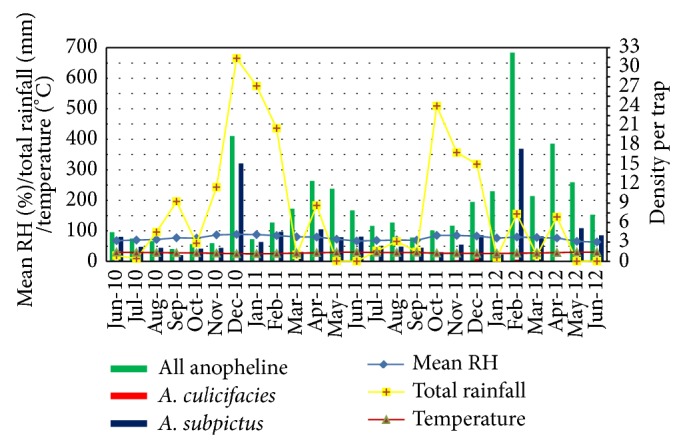
Density of* A. culicifacies*,* A. subpictus*, and all anophelines collected by CBHC with climatic variables.

The outdoor resting anopheline density by CBHC peaked approximately one to two months after that of the indoor resting population. The highest* A. subpictus* density was observed in February 2012 (Figure 6).* A. subpictus* density by CBHC was low, when the indoor resting by HC was observed to be high.* A. culicifacies* was found to be fewer in numbers. It was recorded only from October 2011 to April 2012 throughout the study period, where the highest density was detected in December 2011.

#### 3.5.5. Larval Surveillance (LS)

**Figure 7:**
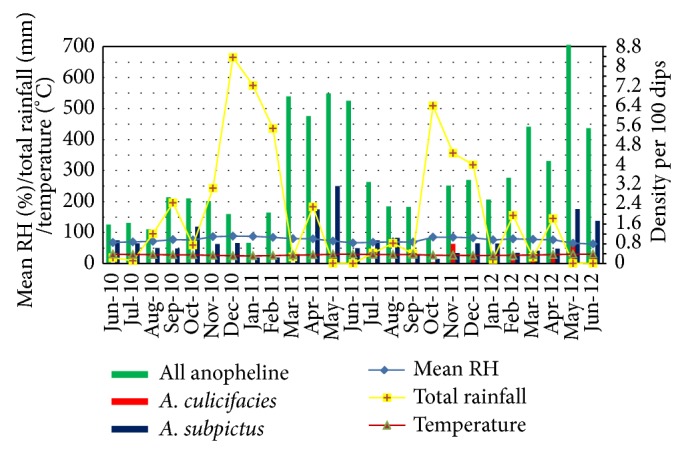
Density of* A. culicifacies*,* A. subpictus*, and all anophelines collected by LS with climatic variables.

The larval density of all anophelines was high during February to May in both 2011 and 2012, approximately one to three months after heavy rains were received in December 2010 and October 2011, respectively (Figure 7). A similar pattern was not identified for* A. subpictus* densities, where the higher densities were found in March to May in both years.* A. culicifacies* was detected continuously since May 2011 to June 2012 except in January 2012. The highest density was detected in June 2012.

## 4. Discussion

The abundance of malaria vector* Anopheles* mosquitoes has not been studied in some parts of Sri Lanka, especially in Northern and Eastern Provinces over the past 30 years because of the security situations. Mosquito species may have shifted their niche with changing weather patterns and ecology in order to attain a wide dissemination in the environment.

The current investigation was focused on the study of malaria vectors and their abundance in selected areas in Trincomalee District. A total of 17 anopheline species were recorded in the study areas. Vector incrimination studies done for most of these anopheline species under experimental laboratory and field conditions have been reported to play a role in malaria transmission in Sri Lanka [15]. Hence, the study of their abundance becomes important for implementation of effective malaria control measures.

*Anopheles subpictus*, the predominant species in all collection techniques we carried out including intradomestic habitats, was the only anopheline species recorded throughout the study period in indoor habitats. Although* A. subpictus* has been incriminated only as a secondary vector of malaria in Sri Lanka [16], given its relative abundance in intra- and peridomestic habitats, it is possible that it plays a more dominant role as a primary vector in the transmission of malaria in Sri Lanka.

The outdoor resting anopheline population demonstrated two distinctive peaks corresponding to periods following monsoonal rains. About 47% and 5% of collections from CBHT and CBNT, respectively, were* A. subpictus*. In cattle-baited net trap collections,* Anopheles peditaeniatus* (43%) and* Anopheles nigerrimus* (31%) were observed; they were not encountered among the indoor resting populations, indicating their exclusive exophilic nature. The presence of* A. nigerrimus* in these areas might contribute to malaria transmission since this species is considered as a secondary vector for malaria. However, indoor residual spraying of insecticides (IRS) is commonly used as the major malaria control intervention targeting adult mosquitoes. Therefore, the tendency of resting adult vector mosquitoes in outdoor resting surfaces will depreciate the effectiveness of IRS as a controlling measure. Hence, the health authorities need to be vigilant to prevent any future epidemics of malaria in these areas.

Cattle-baited trap collections captured all anopheline species prevalent in a particular area. This technique may be good to study the general mosquito abundance in an area especially in monitoring vector species during a malaria elimination phase as in Sri Lanka and even thereafter. The results revel that the mosquitoes collected from CBNT were approximately five times than those of CBHT. The possible reason for this observation may be the fact that since the CBHT is a fixed one at a permanent place, the collection may vary with the seasonality. However, the CBNT is a mobile trap; therefore, during the study period the net traps were placed in different locations proximity to breeding sites and resting places of adult mosquitoes.

The seasonal distribution of anopheline varies in time and space depending upon environmental conditions and availability of breeding habitats. Climatic factors are the most accepted microecological factors that affect mosquito populations. These include temperature, precipitation, and relative humidity [17]. Climate predicts, to a large extent, the natural distribution of malaria [18].

The minimal larval breeding in the district was observed, when rainfall was above 400 mm a month probably due to flushing of larvae as a result of high levels and rapid flow of water in streams and rivers and subsequent flooding that may have led to larval deaths due to reduction in oxygen tension causing physical harm to the larvae [19].

Larvae of* Anopheles* mosquitoes have been found in aquatic bodies such as rice fields, the edges of streams and rivers, and small temporary rain pools. Many species prefer habitats with vegetation, while some breed in open, sunlit pools. The frequency of larval occurrence varied considerably in different habitats. In this study, all anopheline species were reported breeding in tank margins.* Anopheles subpictus* was the predominant species indicating the presence in all 21 habitat categories. There was no habitat found to have larvae of a single anopheline species. Some species such as* Anopheles annularis*,* Anopheles varuna*,* Anopheles tessellatus*,* Anopheles barbirostris*,* Anopheles barbumbrosus*,* Anopheles pallidus*, and* Anopheles pseudojamesi* were limited to selective breeding habitats. This pattern of larval distribution has been attributed to the specificity of mosquito species to prefer different degrees of physicochemical properties of larval habitats [20].

Interestingly,* Anopheles culicifacies*,* A. subpictus*, and some potential vectors were encountered in a variety of breeding habitats including blocked drains with wastewater having low dissolved oxygen levels (<3 mg/L). This has serious implications on the epidemiology of malaria in general and the application of control measures in the country in particular, which have focused on rural populations based on the bioecology of the vector. Rapid unplanned urbanization observed in many parts of the country is changing the context of human population settlements and natural ecosystem interactions that may have contributed to adaptation of anopheline breeding sites that we observed in this study.

The evidence of the adaptation and survival of* A. culicifacies* and* A. subpictus* in polluted water should be a warning signal of the potential for the emergence of urban malaria in Sri Lanka, a phenomenon that has not been reported yet. This needs to be seriously considered by malaria control authorities as the majority of malaria cases reported recently in the country are imported cases detected in people in urban areas. Moreover, adaptation of anophelines to breed in polluted water in urban areas could be a serious concern when* A. stephensi* plays an important role in transmitting urban malaria in neighboring Southern India.

During this study, there was minimal variation in temperature and humidity. The mean monthly temperature ranged between 25.0°C and 30.4°C; the upper limit of RH was over 62% (62.2%–88.5%). The minimal variation in temperature and humidity may also have contributed to the lack of relationship between the abundance of anopheline species and climate variables.

The fact that this study could not sample all possible resting or breeding sites of anophelines in the different localities is acknowledged; our assessment of overall anopheline abundance is based on obtaining representative samples from the selected sampling sites. Mosquito collections were done at monthly intervals. It is possible that rapid changes in weather conditions and availability of breeding sites within the month may have influenced our results. The association between rainfall and density of vectors may have been confounded by changes in the environment affecting the mosquito population and application and effectiveness of malaria control interventions in these areas.

Only a few mosquito species recorded in the country have been incriminated as malarial vectors. It is important to monitor the density of these species, both indoors and outdoors, as well as their breeding habitats for application of vector control measures as Sri Lanka is on the threshold of malaria elimination.

A key point from these results is the potential impact of other mosquito species mainly* A. subpictus* and* A. nigerrimus* on malaria transmission. This survey makes an important contribution in assessing relative abundances of mosquito vectors which may not be considered in malaria control intervention and may point to ways in which mosquito control could be targeted to address the transmission potential of these vectors. Additionally,* A. culicifacies* may actually contribute a relatively small amount of transmission given its low abundance, which again is an important point for effective vector control under the current malaria elimination program in Sri Lanka.

## 5. Conclusions

Routine surveys for mosquito should be an ongoing function of every mosquito controlling program. It is important to determine the abundance, distribution, biology, and relationship with climatic factors of main and secondary malaria vectors in Sri Lanka in order to apply efficient controlling programs under the current malaria elimination program in Sri Lanka.

## Figures and Tables

**Table 1:** *Anopheles* collected from June 2010 to June 2012.

Sampling technique	Unit	Total number of units	Total number collected	Percentage (%)
LS	Dip	598,046	21,347	24.34
HC	Houses	20,694	10,626	12.11
WTC	Trap	3,560	1,790	2.04
CBHC	Trap	1,620	11,622	13.25
CBNC	Trap	1,621	42,325	48.25

Total			87,710	100

**Table 2:** Relative abundance of anophelinesby sampling technique in the Trincomalee District.

*Anopheles* species	Number and percentage (%) of mosquitoes collected by				
	CBHC	CBNC	WTC	HC	LS
*A. aconitus *	5 (0.04)	17 (0.04)	—	—	53 (0.25)
*A. aitkeni *	4 (0.03)	—	—	—	—
*A. annularis *	600 (5.16)	1612 (3.81)	14 (0.78)	19 (0.18)	1038 (4.86)
*A. barbirostris *	564 (4.85)	3976 (9.39)	63 (3.52)	203 (1.91)	1975 (9.25)
*A. barbumbrosus *	17 (0.15)	311 (0.73)	5 (0.28)	13 (0.12)	278 (1.3)
*A. culicifacies *	94 (0.81)	8 (0.02)	4 (0.22)	2 (0.01)	1068 (5.0)
*A. jamesii *	138 (1.19)	764 (1.81)	1 (0.06)	—	79 (0.37)
*A. karwari *	—	3 (0.007)	—	—	—
*A. maculatus *	2 (0.02)	—	—	—	14 (0.07)
*A. nigerrimus *	1774 (15.26)	12938 (30.57)	217 (12.12)	452 (4.25)	5267 (24.67)
*A. pallidus *	521 (4.48)	808 (1.91)	26 (1.45)	60 (0.56)	1430 (6.69)
*A. peditaeniatus *	1209 (10.4)	18185 (42.97)	105 (5.86)	212 (1.99)	3109 (14.56)
*A. pseudojamesi *	71 (0.61)	430 (1.02)	—	—	81 (0.38)
*A. subpictus *	5419 (46.6)	2104 (4.97)	1143 (63.85)	9158 (86.18)	5278 (24.72)
*A. tessellatus *	38 (0.33)	88 (0.21)	1 (0.05)	4 (0.04)	52 (0.24)
*A. vagus *	1087 (9.35)	1052 (2.48)	195 (10.95)	473 (4.45)	980 (4.59)
*A. varuna *	79 (0.68)	29 (0.07)	15 (0.84)	30 (0.28)	645 (3.02)

Total	11622 (100)	42325 (100)	1790 (100)	10626 (100)	21347 (100)

**Table 3:** Density of *A. culicifacies*, *A. subpictus,* and all anopheline collected by different surveillance techniques.

Month and year	Climatic data			Density by each technique														
	Mean Tem (°C)	Mean RF (mm)	Mean RH (%)	CBHT (per trap)			CBNT (per trap)			WTC (per trap)			HC (per house)			LS (per 100 dips)		
				*A. culicifacies *	*A. subpictus *	All anopheline	*A. culicifacies *	*A. subpictus *	All anopheline	*A. culicifacies *	*A. subpictus *	All anopheline	*A. culicifacies *	*A. subpictus *	All anopheline	*A. culicifacies *	*A. subpictus *	All anopheline
Jun-10	30.1	19.30	67.9	0.00	3.78	4.50	0.00	0.37	7.98	0.00	0.42	0.63	0.00	0.42	0.63	0.005	0.94	1.58
Jul-10	29.2	9.14	70.0	0.00	2.26	3.52	0.00	0.81	48.58	0.00	0.23	0.30	0.00	0.23	0.30	0.005	0.82	1.64
Aug-10	28.7	96.02	71.9	0.00	2.09	3.13	0.00	0.79	9.73	0.00	0.11	0.24	0.00	0.11	0.24	0.000	0.63	1.39
Sep-10	28.0	196.1	77.2	0.00	0.96	1.92	0.00	0.87	7.36	0.00	0.14	0.24	0.00	0.14	0.24	0.005	0.61	2.69
Oct-10	28.0	59.44	76.2	0.00	1.97	2.65	0.00	1.32	4.31	0.00	0.10	0.14	0.00	0.10	0.14	0.00	1.50	2.64
Nov-10	26.6	243.4	86.7	0.00	2.09	2.81	0.00	1.09	7.46	0.00	0.09	0.10	0.00	0.09	0.10	0.00	0.79	2.53
Dec-10	25.2	665.2	88.5	0.00	15.14	19.35	0.00	2.86	17.11	0.00	0.28	0.42	0.00	0.28	0.42	0.00	0.83	2.01
Jan-11	25.0	574.5	87.8	0.00	3.00	3.43	0.00	1.40	22.47	0.00	0.24	0.35	0.00	0.24	0.35	0.00	0.25	0.84
Feb-11	25.9	436.1	84.6	0.00	4.58	6.00	0.00	1.78	18.66	0.00	0.19	0.43	0.00	0.19	0.43	0.00	0.25	2.07
Mar-11	27.5	21.85	80.4	0.00	1.45	8.14	0.00	3.42	73.01	0.00	0.12	0.21	0.00	0.12	0.21	0.00	0.38	6.78
Apr-11	28.2	183.1	79.9	0.00	4.94	12.42	0.00	2.51	38.02	0.00	0.79	0.85	0.00	0.79	0.85	0.00	2.20	5.98
May-11	29.9	1.01	72.7	0.00	3.74	11.22	0.00	3.70	20.27	0.00	1.74	1.89	0.00	1.74	1.89	0.28	3.14	6.90
Jun-11	30.1	0.51	66.7	0.00	3.80	7.87	0.00	1.72	19.78	0.00	0.23	0.34	0.00	0.23	0.34	0.02	0.62	6.60
Jul-11	29.3	34.04	68.5	0.00	2.38	5.49	0.00	0.48	45.41	0.00	0.22	0.33	0.00	0.22	0.33	0.008	0.94	3.31
Aug-11	29.1	65.54	70.1	0.00	2.28	5.99	0.00	0.43	32.79	0.00	0.04	0.10	0.00	0.04	0.10	0.04	1.04	2.32
Sep-11	29.4	31.75	69.4	0.00	2.11	3.67	0.00	0.11	8.59	0.00	0.05	0.09	0.00	0.05	0.09	0.04	0.35	2.30
Oct-11	27.0	509.0	85.2	0.02	1.42	4.78	0.00	0.49	10.55	0.02	0.02	0.04	0.00	0.02	0.04	0.06	0.19	1.01
Nov-11	26.7	356.4	84.9	0.18	2.60	5.51	0.01	1.33	20.01	0.00	0.26	0.30	0.00	0.26	0.30	0.80	0.43	3.16
Dec-11	26.1	318.5	83.0	0.43	4.05	9.20	0.10	1.78	56.54	0.00	0.55	0.60	0.001	0.55	0.60	0.36	0.82	3.39
Jan-12	26.3	12.70	76.4	0.34	1.07	10.84	0.00	0.16	62.11	0.00	1.40	1.42	0.001	1.40	1.42	0.00	0.81	2.59
Feb-12	26.8	155.2	79.8	0.28	17.38	32.23	0.00	0.16	34.70	0.00	0.01	0.03	0.00	0.01	0.03	0.14	0.44	3.48
Mar-12	27.8	20.32	78.0	0.07	3.86	10.09	0.00	0.20	10.27	0.00	0.15	0.16	0.00	0.15	0.15	0.40	0.52	5.55
Apr-12	28.7	145.5	76.7	0.07	0.00	18.19	0.00	2.32	8.83	0.00	0.74	0.75	0.00	0.74	0.75	0.29	0.60	4.16
May-12	30.4	0.51	65.6	0.00	5.14	12.22	0.00	2.29	6.82	0.00	2.21	2.23	0.00	2.21	2.23	0.86	2.21	8.87
Jun-12	30.2	0.76	63.4	0.00	4.02	7.20	0.00	1.08	5.81	0.00	0.36	0.37	0.00	0.36	0.37	0.36	1.73	5.49

**Table 4:** Diverse breeding habitats of *An. culicifacies* and other anophelinespecies in Trincomalee District.

Breeding habitats	Species														
	*An. culicifacies *	*An. subpictus *	*An. peditaeniatus *	*An. pallidus *	*An. vagus *	*An. varuna *	*An. barbirostris *	*An. annularis *	*An. aconitus *	*An. jamesii *	*An. nigerrimus *	*An. barbumbrosus *	*An. tessellatus *	*An. pseudojamesi *	*An. maculatus *
Tank margin	+	+	+	+	+	+	+	+	+	+	+	+	+	+	
Lake margin		+	+	+	+	+	+	+		+	+	+	+		
Canal with vegetation	+	+	+		+	+	+				+				
Marshy land	+	+	+	+	+	+	+	+	+	+	+				
Field canal	+	+	+	+	+	+	+	+	+		+				
Main canal	+	+	+	+	+	+	+	+	+	+	+			+	
Paddy field	+	+	+	+	+	+	+	+		+	+	+		+	
Pond	+	+	+	+	+	+	+	+		+	+	+	+		
Rock pool	+	+	+	+	+	+	+	+		+	+				
Earth well (domestic)	+	+	+	+	+	+	+	+		+	+	+	+		
Built well (domestic)	+	+	+	+	+	+	+	+	+	+	+	+			
Common well		+													
Agricultural well		+					+								
Burrow pit	+	+	+	+	+	+	+	+	+	+	+	+	+		+
Animal foot print		+	+	+	+		+				+	+			
Rain water pool	+	+	+	+	+	+	+	+	+	+	+		+	+	+
Quarry pit	+	+	+	+	+	+	+	+	+	+	+				
River Margin		+	+	+	+		+	+			+				
Tyre mark		+	+	+	+		+								
Waste water collection	+	+	+	+	+		+	+			+				
Water storage tank	+	+	+				+	+		+	+				

**Table 5:** Correlation between anopheline densities climate variables.

Lag period (Months)	Pearson correlation coefficient (*P* value) between vector densities and climatic factors								
	Rainfall			Temperature			Humidity		
	All Anopheline	*A. culicifacies *	*A. subpictus *	All Anopheline	*A. culicifacies *	*A. subpictus *	All Anopheline	*A. culicifacies *	*A. subpictus *
Hand collection									
0	−0.248 (0.232)	−0.001 (0.996)	−0.268 (0.195)	0.279 (0.177)	−0.332 (0.105)	0.279 (0.177)	−0.267 (0.197)	−0.267 (0.197)	0.129 (0.538)
1	0.087 (0.685)	0.252 (0.235)	0.052 (0.811)	−0.064 (0.767)	−0.290 (0.168)	−0.045 (0.836)	0.236 (0.267)	0.210 (0.325)	0.298 (0.157)
2	−0.007 (0.973)	0.394 (0.063)	−0.030 (0.893)	−0.216 (0.323)	−0.195 (0.373)	−0.206 (0.345)	0.319 (0.137)	0.300 (0.165)	0.345 (0.107)

Window trap									
0	0.315 (0.126)	0.358 (0.079)	0.297 (0.149)	−0.269 (0.194)	−0.128 (0.542)	−0.205 (0.326)	0.236 (0.255)	0.200 (0.338)	0.242 (0.245)
1	0.402 (0.052)	−0.150 (0.484)	0.347 (0.097)	−0.344 (0.099)	0.196 (0.357)	−0.244 (0.251)	0.455 (0.026)	0.399 (0.054)	−0.226 (0.289)
2	0.206 (0.346)	−0.124 (0.572)	0.089 (0.685)	−0.317 (0.141)	0.181 (0.408)	−0.210 (0.337)	0.487 (0.019)	0.389 (0.067)	−0.233 (0.284)

Cattle-baited trap									
0	0.118 (0.575)	0.066 (0.754)	0.310 (0.132)	−0.257 (0.214)	−0.415 (0.039)	−0.278 (0.178)	0.212 (0.308)	0.220 (0.290)	0.252 (0.224)
1	−0.087 (0.686)	0.230 (0.280)	−0.034 (0.875)	−0.375 (0.071)	−0.403 (0.051)	−0.292 (0.166)	0.272 (0.199)	0.231 (0.277)	0.340 (0.104)
2	0.225 (0.303)	0.362 (0.090)	0.098 (0.658)	−0.43 (0.037)	−0.252 (0.245)	−0.246 (0.258)	0.368 (0.084)	0.211 (0.334)	0.314 (0.145)

Cattle-baited net									
0	−0.134 (0.524)	0.187 (0.370)	0.156 (0.457)	−0.217 (0.296)	−0.266 (0.199)	−0.034 (0.872)	0.092 (0.661)	0.203 (0.331)	0.214 (0.304)
1	0.232 (0.275)	0.246 (0.247)	0.290 (0.169)	−0.282 (0.181)	−0.178 (0.405)	−0.262 (0.216)	0.239 (0.260)	0.499 (0.013)	0.269 (0.204)
2	0.598 (0.003)	0.329 (0.125)	0.287 (0.185)	−0.433 (0.039)	−0.080 (0.716)	−0.415 (0.049)	0.539 (0.008)	0.460 (0.027)	0.204 (0.350)

Larval collection									
0	−0.248 (0.232)	−0.001 (0.996)	−0.268 (0.195)	0.279 (0.177)	−0.332 (0.105)	0.279 (0.177)	−0.267 (0.197)	−0.267 (0.197)	0.129 (0.538)
1	0.087 (0.685)	0.252 (0.235)	0.052 (0.811)	−0.064 (0.767)	−0.29 (0.168)	−0.045 (0.836)	0.236 (0.267)	0.210 (0.325)	0.298 (0.157)
2	−0.007 (0.973)	0.394 (0.063)	−0.030 (0.893)	−0.216 (0.323)	−0.195 (0.373)	−0.206 (0.345)	0.319 (0.137)	0.300 (0.165)	0.345 (0.107)

*P* values in parentheses.
